# Differential expression of *HIF1A* and its downstream target *VEGFA* in the main subtypes of renal cell carcinoma and their impact on patient survival

**DOI:** 10.3389/fonc.2023.1287239

**Published:** 2023-11-20

**Authors:** Ante Strikic, Josipa Kokeza, Marin Ogorevc, Nela Kelam, Martina Vukoja, Petar Dolonga, Sandra Zekic Tomas

**Affiliations:** ^1^Department of Oncology and Radiotherapy, University Hospital of Split, Split, Croatia; ^2^Department of Pulmonology, University Hospital of Split, Split, Croatia; ^3^Department of Anatomy, Histology and Embryology, University of Split School of Medicine, Split, Croatia; ^4^Laboratory of Morphology, Department of Histology and Embryology, School of Medicine, University of Mostar, Mostar, Bosnia and Herzegovina; ^5^University of Split School of Medicine, Split, Croatia; ^6^Department of Pathology, Forensic Medicine and Cytology, University Hospital of Split, Split, Croatia; ^7^Department of Pathology, University of Split School of Medicine, Split, Croatia

**Keywords:** HIF-1α, VEGF, VEGF-A, renal carcinoma, renal cell carcinoma

## Abstract

Renal cell carcinoma (RCC) represents around 3% of all cancers, with the most frequent histological types being clear-cell RCC (ccRCC), followed by papillary (pRCC) and chromophobe (chRCC). Hypoxia-inducible factors (HIFs), which promote the expression of various target genes, including vascular endothelial growth factor (VEGF) and the high- affinity glucose transporter 1, have an important role in the pathogenesis of RCC. This study investigated the immunohistochemical expression of HIF-1α and VEGF-A, showing significantly higher HIF-1α nuclear expression in pRCC compared to ccRCC, while there was no significant difference in VEGF-A protein expression between the analyzed histological RCC subtypes. The quantitative reverse transcription polymerase chain reaction for *HIF1A* showed no statistical difference between histological types. Data from publicly available RNA sequencing databases were analyzed and showed that, compared to healthy kidney tissue, *VEGFA* was significantly up-regulated in ccRCC and significantly down-regulated in pRCC. The comparison between histological subtypes of RCC revealed that *VEGFA* was significantly up-regulated in ccRCC compared to both pRCC and chRCC. There was no statistically significant difference in survival time between *HIF1A* high- and low-expression groups of patients. As for *VEGFA* expression, pRCC patients with low expression had a significantly higher survival rate compared to patients with high *VEGFA* expression.

## 1 Introduction

Renal cell carcinoma (RCC) is a relatively common type of cancer, with an estimated 431,288 new cases of kidney cancer and 179,368 deaths from the disease worldwide in 2020 (1). According to the American Cancer Society, the five-year relative survival rate for all stages of kidney cancer is around 75%; however, the survival rates vary significantly. In localized kidney cancer, the five-year survival rate is 93% compared to cases where the cancer has spread to distant organs with 12% (2).

The 2022 WHO classification system recognizes several types of renal tumors, including clear-cell RCC (ccRCC), papillary RCC (pRCC), oncocytic and chromophobe renal tumors (chRCC), collecting duct tumors, other renal tumors and molecularly defined renal carcinomas, metanephric tumors, mixed epithelial and stromal renal tumors, renal mesenchymal tumors, embryonal neoplasms of the kidney, and miscellaneous renal tumors (3).

The most important genetic mutation in renal cancer tumorigenesis is the mutation of the von Hippel Lindau gene (*VHL*), a tumor suppressor gene encoding the von Hippel Lindau protein (pVHL) (4). The primary function of pVHL is to target and degrade hypoxia-inducible factors (HIFs), particularly HIF-1α and HIF-2α, under normal oxygen conditions. HIFs are transcription factors that regulate genes involved in oxygen sensing, angiogenesis, and cell growth (5). In ccRCC, the *VHL* gene is often mutated or inactivated, resulting in loss of function of pVHL (6). This leads to the stabilization and accumulation of HIF-1α and HIF-2α, even under normoxic conditions. Stabilized HIFs promote the expression of various target genes, including vascular endothelial growth factor (*VEGFA*) and platelet-derived growth factor (*PDGF*). VEGF-A is a potent pro-angiogenic factor that stimulates the growth of new blood vessels from existing ones, a process called angiogenesis (7, 8). In RCC, VEGF is often overexpressed, leading to excessive angiogenesis, which contributes to tumor growth and metastasis (9). VEGF-A has been particularly associated with ccRCC, where its overexpression is commonly observed. Other RCC subtypes, such as papillary and chromophobe RCC, may also show VEGF-A involvement, although to a lesser extent (10).

The present study aimed to determine *HIF1A* mRNA expression, the protein expression of HIF-1α and VEGF-A in the three most common histological types of renal cancer – ccRCC, pRCC, chRCC, as well as to analyze *HIF1A* and *VEGFA* differential gene expression and its influence on patient’s survival.

## 2 Materials and methods

### 2.1 Tissue procurement and processing

This retrospective cross-sectional study was conducted in the Pathology Department, University Hospital Centre Split, Croatia, and was approved by the Hospital Ethics Committee (class: 500-03/20-01/09, approval number: 2181-147-01/06/M.S.-20-09, approval date: 13 May 2020). The tumor samples were selected from the Pathology Department’s archive. The institute’s database of pathohistological reports was searched using the International Classification of Diseases (ICD)-10 code: C64, which stands for the malignant neoplasm of the kidney. The inclusion criteria were operational materials (radical or complete nephrectomies) with patients’ medical history available for the clinical data. Immediately after surgery the specimens were analyzed and sectioned by a genitourinary pathologist, after which the sections were immediately submerged into a 4% buffered formalin solution and further processed by standard protocols. The samples from biopsies or the ones with insufficient clinical data were excluded from the study. A total of 39 paraffin blocks containing RCC samples were collected, 14 of those being RCCs, 13 pRCCs, and 12 chRCCs. The tumor samples included in the study were classified according to the 2022 World Health Organization Classification of Tumors of the Urinary System and Male Genital Organs. Although nuclear grading is not recommended for all RCC histological subtypes, for the purpose of this study we’ve assigned nuclear grade to each RCC sample with the use of the WHO/ISUP grading system (3). From each paraffin block, a 4 μm-thick section was cut, mounted, and dried at 37°C. Then, the sections were stained with hematoxylin and eosin and reevaluated by a genitourinary pathologist.

### 2.2 Immunohistochemical staining and HSCORE calculation

HIF-1α and VEGF-A immunohistochemical analyses were done as described in our previous work (11). Briefly, sections from RCC paraffin blocks were placed on super frost glass (Thermoscientific, Germany) and processed in an automatic stainer (Ventana Bench Mark Ultra Autostainer, Ventana Roche, Tucson, Arizona, USA). The following antibodies were used: primary rabbit polyclonal IgG antibody clone H-206 (Santa Cruz Biotechnology Inc., Dallas, Texas, USA) for HIF-1α and primary polyclonal rabbit antibody clone ab46154 (Abcam, England) for VEGF-A. Ultra view Universal DAB detection kit (Ventana, Tucson, Arizona, USA) was used as a secondary antibody. Nuclear brown staining was considered positive for HIF-1α and brown cytoplasmic and membranous staining was considered positive for VEGF-A. Placenta tissue served as a positive control. The HIF-1α protein expression was determined as the percentage of tumor cells displaying nuclear positivity. The VEGF-A protein expression was determined using the HScore method according to the following formula HScore=Σ Pi (i + 1), where i=staining intensity determined as 1 (weak), 2 (moderate), or 3 (strong), and Pi is the percentage of staining of kidney cancer cells for each intensity (12). For each RCC sample, 10 high- power fields of view (HPF) were analyzed. HScore was determined for each visual field of the sample. The final HScore for each sample was calculated as the arithmetic mean of all 10 HPF.

### 2.3 RNA Isolation and Reverse transcription

Total RNA was isolated from 20 human formalin-fixed paraffin-embedded kidney samples (clear cell n=7; chromophobe n=6; papillary n=7). Each sample contained 5 mm2 of tumor tissue without necrosis and normal kidney tissue. Multiple 8 µm thick tissue slices were placed in RNAse-free tubes and processed with High Pure RNA Paraffin (Cat. No. 03270289001; Roche, Basel, Switzerland) according to the manufacturer’s instructions. The protocol starts with deparaffinization of the tissue embedded in paraffin, washing in absolute ethanol, and centrifuging at maximum speed for 2 min. Proteinase K and the Tissue Lysis Buffer were added to the dried pellet for digestion and incubated overnight. The next day, the Binding buffer and ethanol were added to the lysate, and the solution was applied to a spin column. The bound RNA was washed from the column. DNase working solution and Incubation buffer were added to the eluate and mixed. The Qubit™ 4 Fluorometer (Thermo Fisher Scientific Inc., Waltham, MA, USA) was used to quantify the total RNA in each sample. The samples were diluted to match the lowest measured concentration (5.30 ng/µL). 5.30 nanogram of total RNA was reverse transcribed into complementary DNA (cDNA) with a High-Capacity Reverse Transcriptase Kit (Applied Biosystems, CA, USA) using random primers according to the manufacturer’s instructions. cDNA (final volume of 20 µL) was stored at −80°C for subsequent quantification of genes of interest.

#### 2.3.1 qPCR

qPCR analysis was performed using Taqman® Fast Advanced Universal Master Mix II (Applied Biosystems, Waltham, MA, USA) comprising AmpEraseuracil-N-glycosylase and the passive reference dye ROX. ProbesTaqman® gene expression assays for human HIF1A were supplied by Applied Biosystems (Hs00153153_m1). Glyceralde-hyde-3-phosphate dehydrogenase (*GAPDH*) was analyzed as a housekeeping gene (Hs99999905_m1). Taqman real-time PCR was performed using a 2µL cDNA template, 1 µLTaqman® (Applied Biosystems, Waltham, MA, USA) gene expression assay, and 10 µLTaqman® (Applied Biosystems, Waltham, MA, USA) universal master mix (to the final volume of 20 µL of). The PCR protocol used involved heating for 2 min at 50°C for uracil-N-glycosylase activation, then heating for 2 min at 95°C for polymerase activation, followed by 40 cycles of amplification (3 s at 95°C and 30 s at 60°C). We performed duplicate PCRs per gene per cDNA sample. A negative control containing nuclease-free water instead of a cDNA template was used in each experiment. The 2−ΔΔCt method was used as the method of relative quantification. The plate was then analyzed using the Applied Biosystems™ 7500 RT-PCR system (Thermo Fisher Scientific, Waltham, MA, USA). To perform the 2−ΔΔCt method, the average of the ΔCt values from pRCC samples was used as a calibrator to calculate the relative fold gene expression of all samples concerning RCC.

### 2.4 Differential gene expression and survival analysis

The differential expression of the *HIF1A* and *VEGFA* genes between ccRCC, pRCC, chRCC, and healthy kidney tissue was performed using the University of California Santa Cruz (UCSC) Xena platform (http://xena.ucsc.edu/) (13). First, we have selected appropriate studies from the TCGA TARGET Genotype-Tissue Expression (GTEx) study that contains RNAseq data, specifically, the TCGA Kidney Clear Cell Carcinoma (KIRC, n=531), TCGA Kidney Papillary Cell Carcinoma (KIRP, n=289), TCGA Kidney Chromophobe (KICH, n=66), and GTEX Kidney (n=28) studies. A differential expression analysis was performed for each pair of the selected studies. The limma voom method was used for differential expression analysis, with the P-value threshold set at 0.01 and the log2(fold change) threshold set at 1. The differential expression analysis was visualized by volcano plots made in GraphPad Prism 9.0.0. software (GraphPad Software, San Diego, CA, SAD).

The survival analysis was performed as described in our previous work (11). Briefly, we retrieved the data for the RNA expression of the *HIF1A* and *VEGFA* genes and overall survival of patients from the Genomic Data Commons (GDC) TCGA Kidney Clear Cell Carcinoma (KIRC), GDC TCGA Kidney Papillary Cell Carcinoma (KIRP), and GDC TCGA Kidney Chromophobe (KICH) studies using the UCSC Xena platform (http://xena.ucsc.edu/). The data were exported and edited in Microsoft® Excel® 2019 MSO version 2305 (Microsoft Corp., Redmond, WA, USA). After curating the data for double samples, 270 patients were included in the survival analysis for ccRCC, 152 patients for pRCC, and 38 patients for chRCC. Survival analysis was performed based on expression groups (i.e., between the lowest and highest quartile for each gene) in GraphPad 9.0.0. software (GraphPad Software, San Diego, CA, USA). The Log-rank test and Kaplan–Meier method was used for the statistical analysis of the survival length.

### 2.5 Statistical analysis

The statistical analyses were done in GraphPad Prism 9.0.0 software (GraphPad Software, San Diego, CA, USA). We used the Shapiro–Wilk test to determine the normality of the data distribution. Correlations between the patient’s age at the time of diagnosis, tumor size, HIF-1α, and VEGF-A protein expression were performed using Spearman’s rank correlation coefficient. The existence of significant differences in HIF-1α and VEGF-A protein expression between sexes, tumor nuclear grades, and tumor groups based on the presence of necrosis was determined by the Mann–Whitney U test, while the Kruskal–Wallis test with uncorrected Dunn’s *post-hoc* test were used for the differences between tumor histological subtypes. For the *HIF1A* RT−qPCR analysis, Ordinary one-way ANOVA followed by Tukey’s multiple comparison test was performed for statistical analysis. Statistical significance was set at p < 0.05. All data are presented as the mean ± standard deviation.

## 3 Results

The study included a total of 39 patients diagnosed with RCC; 26 men (66.66%) and 13 women (33.33%). The average age for female patients was 66 and for male patients 55 years of age. ccRCC was diagnosed in 14 cases (35.89%), pRCC in 13 cases (33.33%) and 12 cases were chRCC (30,76%). There was no statistically significant difference between studied histological RCC types regarding the patient’s sex. chRCC patients were diagnosed at an earlier age compared to ccRCC and pRCC patients which was statistically significant (p=0.037). The gross and histological characteristics of the studied RCCs are presented in **Table 1**. There was no statistically significant difference in tumor size, pathological stage of the disease according to the primary tumor (pT), presence of sarcomatoid features, and lymphovascular invasion. pRCC had a significantly higher number of cases with tumor necrosis compared to both ccRCC and pRCC (p=0.026).

**Table 1:** The gross and histological characteristics of studied renal cell carcinomas (RCC); clear cell RCC (ccRCC), papillary RCC (pRCC) and chromophobe RCC (chRCC).

	ccRCC *n=14*	pRCC *n=13*	chRCC *n=12*	P
Tumor size (cm)	7 ± 3.8	5 ± 3	4 ± 2.3	0.124^‡^
Primary tumor (pT)^†^				
pT1a pT1b pT2a pT2b pT3a	4 3 0 1 6	5 3 2 0 3	8 3 0 0 1	0.194*
Sarcomatoid Features	2	1	1	0.824*
Tumor necrosis	3	8	1	0.026*
Lymphovacular Invasion	2	2	0	0.948*

^‡^Kruskal-Wallis test with Dunn’s post-hoc test; *Chi-squared test; ^†^pT1a Tumor less than or equal to 4 cm in greatest dimension, limited to the kidney; pT1b Tumor greater than 4 cm but less than or equal to 7 cm in greatest dimension limited to the kidney; pT2a Tumor greater than 7 cm but less than or equal to 10 cm in greatest dimension, limited to the kidney; pT2b Tumor greater than 10 cm, limited to the kidney; pT3a Tumor extends into the renal vein or its segmental branches, or invades the pelvicalyceal system, or invades perirenal and/or renal sinus fat but not beyond Gerota’s fascia.

### 3.1 HIF-1α and VEGF-A protein expression

**Figure 1:**
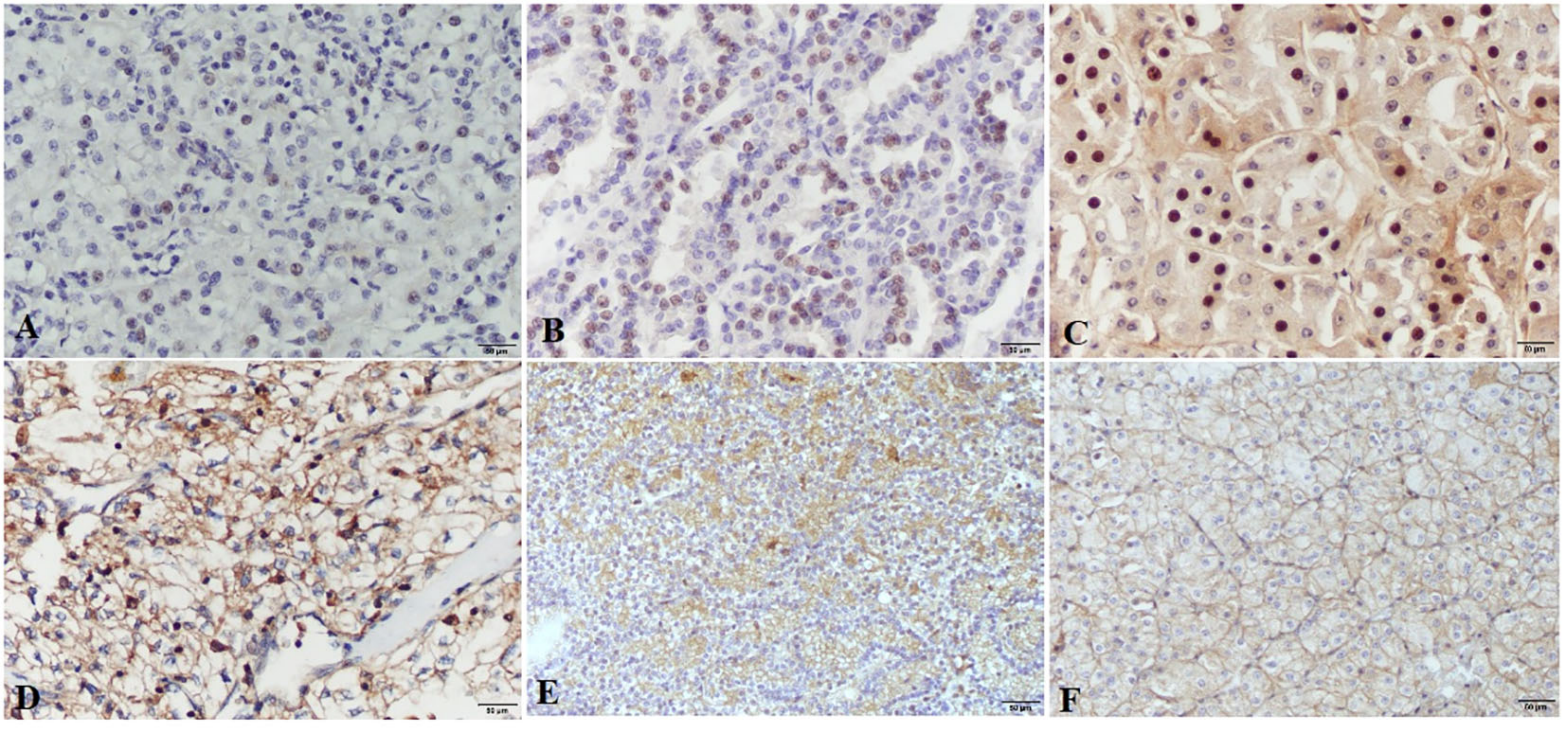
HIF-1α and VEGF-A expression in renal cell carcinomas. HIF-1α nuclear expression is present in clear cell renal carcinomas **(A)**, papillary renal cell carcinomas **(B)** and chromophobe renal cell carcinomas **(C)**, images **(A–C)** are taken at ×200 magnification; scale bars represent 50 µm. VEGF-A shows strong to moderate expression in clear cell renal carcinomas **(D)**, papillary renal cell carcinomas **(E)** and Chromophobe renal cell carcinomas **(F)**, images **(D–F)** are taken at ×100 magnification; scale bars represent 50 µm.

Nuclear staining of tumor cells for HIF-1α was considered positive. The intensity of the nuclear staining ranged from moderate to strong (**Figure 1**). The protein expression of HIF-1α, measured as the percentage of tumor cells displaying nuclear positivity, was not significantly different regarding RCC patients’ sex, type of tumor removal surgery, macroscopic or microscopic tumor extension, clinical stage, nuclear grade, or the presence of tumor necrosis (**Table 2**). There was, however, a significant difference between histological subtypes with pRCC having higher HIF-1α protein expression than ccRCC. There was no significant correlation between HIF-1α protein expression and patients’ age or tumor size.

**Table 2:** Immunohistochemical expression of HIF-1α and VEGF-A in RCC according to clinicopathological characteristics of patients.

	HIF-1α score	P	VEGF-A score	P
Sex				
Male (n=26) Female (n=13)	18.18 ± 13.48 23.87 ± 14.21	0.2279*	1.89 ± 1.23 3.02 ± 1.19	0.0115*
Type of surgery				
Radical nephrectomy (n=27) Partial nephrectomy (n=2) Tumorectomy (n=7)	20.40 ± 14.85 28.43 ± 18.21 20.33 ± 10.39	0.6680^†^	2.28 ± 1.32 2.10 ± 2.69 2.26 ± 1.17	0.9889^†^
Macroscopic tumor extension				
Confined to kidney (n=28) Extends beyond kidney (n=8)	22.09 ± 14.00 16.41 ± 13.84	0.3943*	2.32 ± 1.30 2.06 ± 1.44	0.6737*
Microscopic tumor extension				
Confined to kidney (n=26) Extends beyond kidney (n=10)	23.29 ± 13.80 14.42 ± 12.92	0.1327*	2.42 ± 1.29 1.84 ± 1.37	0.2885*
Clinical stage				
Stage I (n=25) Stage II (n=3) Stage III (n=8)	22.73 ± 13.12 16.78 ± 23.05 16.41 ± 13.84	0.5465^†^	2.38 ± 1.25 1.82 ± 1.93 2.06 ± 1.44	0.7471^†^
Histological subtype				
ccRCC (n=14) pRCC (n=13) chRCC (n=12)	14.72 ± 12.07 28.52 ± 14.78 17.16 ± 10.88	0.0224^†^	1.78 ± 1.21 2.61 ± 1.31 2.45 ± 1.38	0.2744^†^
Nuclear grade (Furham)				
low (n=16) high (n=18)	17.74 ± 16.14 19.52 ± 11.93	0.5449*	1.98 ± 1.32 2.53 ± 1.31	0.2000*
Tumor necrosis				
present (n=12) not present (n=24)	23.29 ± 16.24 18.65 ± 12.66	0.2420*	2.11 ± 1.34 2.33 ± 1.32	0.6899*

*Mann-Whitney U test, ^†^Kruskal-Wallis test with Uncorrected Dunn’s post-hoc test.

VEGF-A expression was determined according to the intensity of membranous and/or cytoplasmatic brown staining of tumor cells as weak, moderate, or strong. Most of the tumors had a heterogeneous staining pattern (**Figure 1**). While VEGF-A protein expression measured by the Hscore was significantly higher in female patients compared to males, there were no significant differences regarding the type of tumor removal surgery, macroscopic or microscopic tumor extension, clinical stage, histological subtype, nuclear grade, or the presence of necrosis. There was also no significant correlation between VEGF-A protein expression and patients’ age or tumor size.

### 3.2 *HIF1A* RT-qPRC analysis

**Figure 2:**
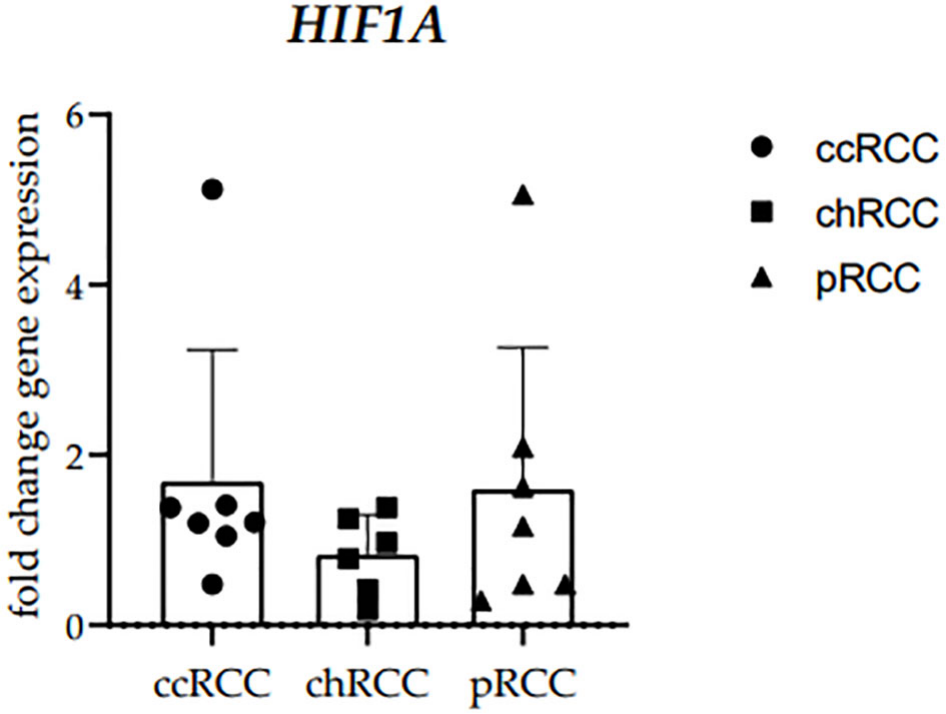
The HIF1A mRNA fold change gene expression comparison between clear cell (ccRCC), chromophobe (chRCC) and papillary (pRCC) renal cell carcinoma. Each dot indicates an individual sample.

The RT−qPCR analysis of human RCCs was performed using the primer for HIF-1α. The *HIF1A* mRNA fold change gene expression comparison between clear cell (ccRCC), chromophobe (chRCC), and papillary (pRCC) renal cell carcinoma was performed and no statistically significant difference was observed between the examined groups (**Figure 2**). *GAPDH* median cycles for ccRCC (32.94), chRCC (31.67), and PRCC (34.88) indicate that the cohorts are comparable (**Supplementary Table S1**).

### 3.3 Differential gene expression

**Figure 3:**
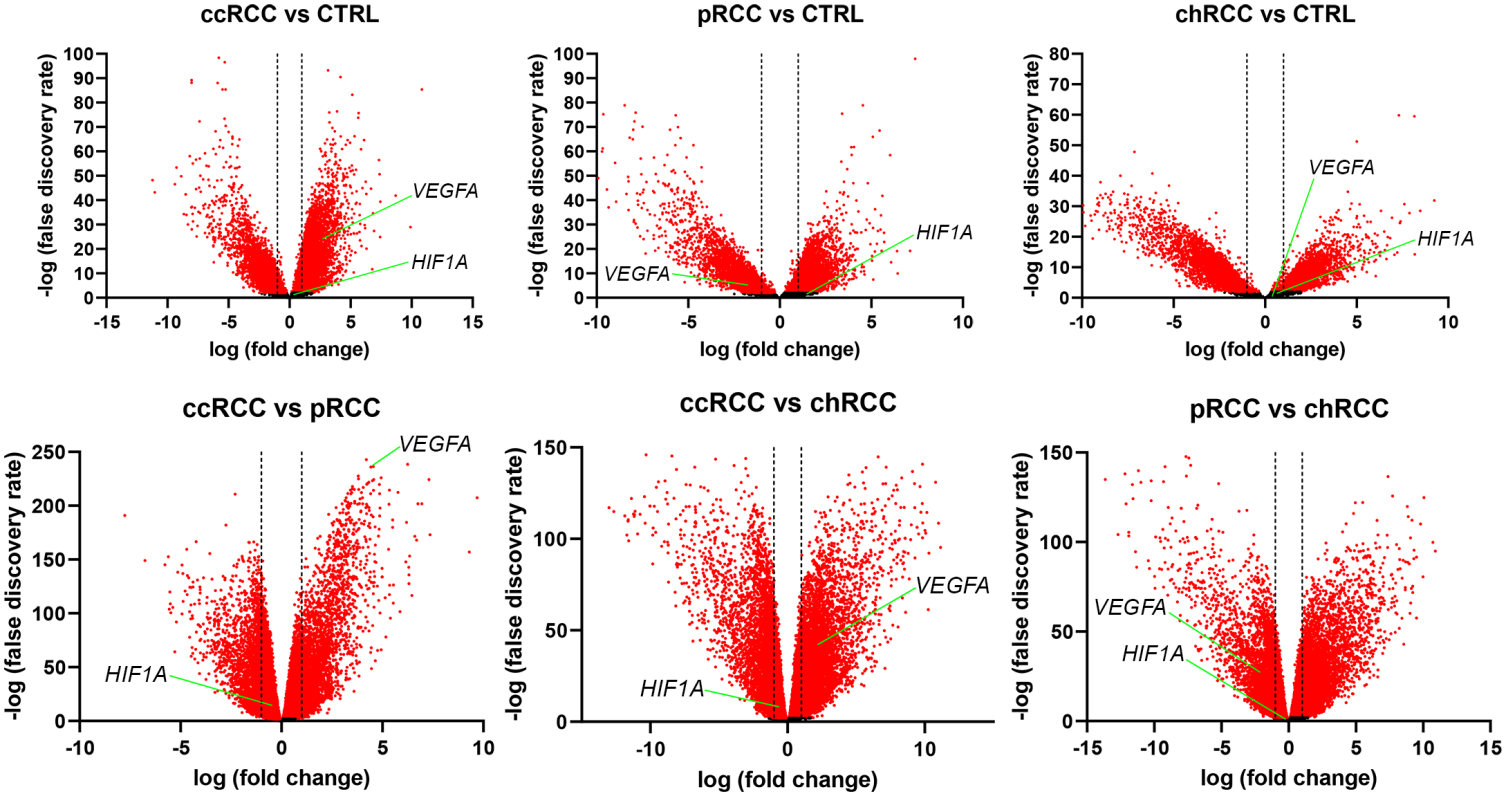
Volcano plots for RCC and healthy kidney tissue. The x-axis represents the base 2 logarithm of fold change, while the y-axis represents the negative base 10 logarithm of the false discovery rate. Each dot on the plots represents a gene. All genes with a –log (false discovery rate)>2, which corresponds to p<0.01, are considered significantly differentially expressed and their dots are colored red, while the other genes’ dots are black. The vertical dashed lines correspond to x=–1 and x=1, which marks a two-fold change. All dots between the dashed lines are not considered differentially expressed. The red dots right of the right dashed line represent significantly up-regulated genes, while those left of the left dashed line are considered significantly down-regulated. The positions of *HIF1A* and *VEGFA* are marked.

The RNA sequencing (RNAseq) data from The Cancer Genome Atlas (TCGA) Kidney Clear Cell Carcinoma (KIRC), TCGA Kidney Papillary Cell Carcinoma (KIRP), TCGA Kidney Chromophobe (KICH), and GTEx Kidney studies was analyzed to determine whether *HIF1A* and *VEGFA* were differentially expressed in the analyzed groups, where a two-fold change was considered significant. There was no differential expression of *HIF1A* between any of the analyzed groups (**Figure 3**). When comparing RCC with normal kidney tissue, *VEGFA* was significantly up-regulated in ccRCC and significantly down-regulated in pRCC, while there was no significant difference between chRCC and normal kidney tissue. The comparison between histological subtypes of RCC revealed that *VEGFA* was significantly up-regulated in ccRCC compared to both pRCC and chRCC. In fact, *VEGFA* was the fourth most up-regulated gene in ccRCC compared to pRCC. *VEGFA* was also significantly downregulated in pRCC compared to chRCC.

### 3.4 *HIF1A* and *VEGFA* expression and patient survival

**Figure 4:**
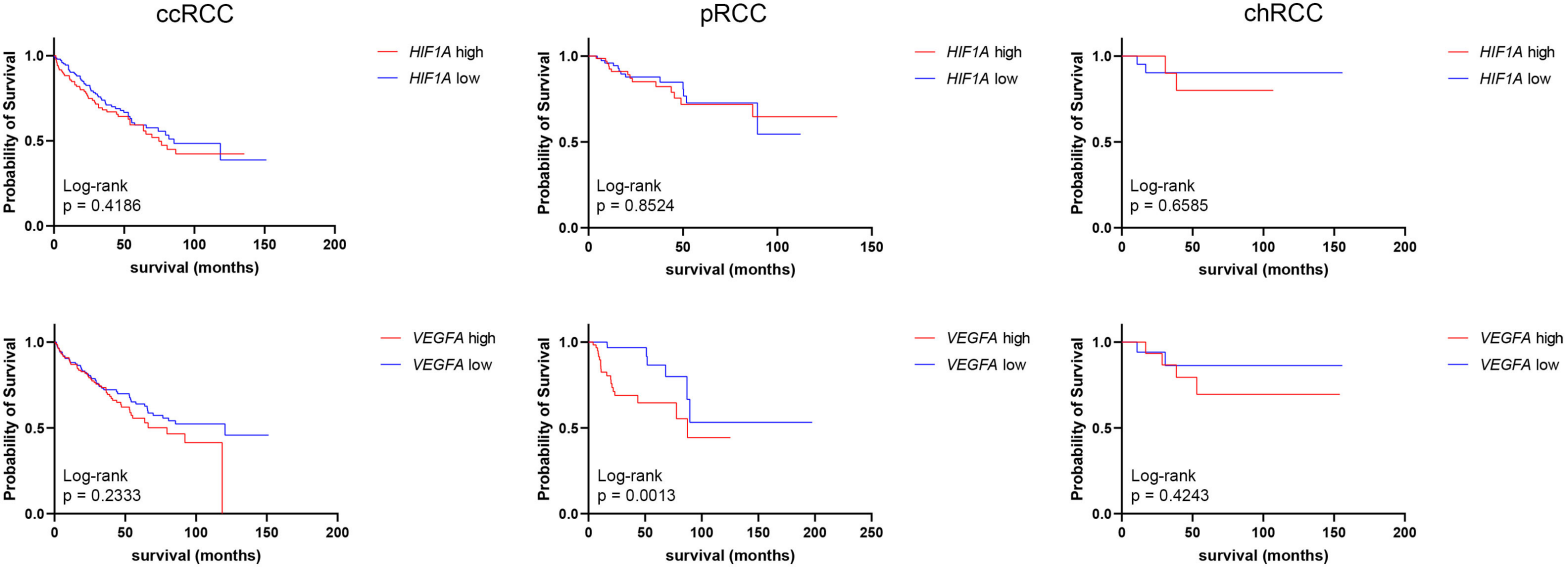
Graphic representation of survival analysis (months) of *HIF1A* and *VEGFA* high (red line)- and low (blue line)-expression in ccRCCs, pRCCs, and chRCCs. The Kaplan–Meier method and Log-rank test were used to determine survival length significance. The data are used from the GDC TCGA Kidney Clear Cell Carcinoma (KIRC), GDC TCGA Kidney Papillary Cell Carcinoma (KIRP), and GDC TCGA Kidney Chromophobe (KICH) studies.

*HIF1A* and *VEGFA* mRNA expressions obtained from the GDC TCGA Kidney Clear Cell Carcinoma (KIRC), GDC TCGA Kidney Papillary Cell Carcinoma (KIRP), and GDC TCGA Kidney Chromophobe (KICH) studies were analyzed to determine the median survival time (mst) and survival rate between low- and high-expression groups (**Figure 4**). There was no statistically significant difference in survival time between *HIF1A* high- and low-expression groups of ccRCC, pRCC, or chRCC patients. As for *VEGFA* expression, there were no significant differences in survival time of ccRCC and chRCC patients based on *VEGFA* mRNA levels, however, pRCC patients with low *VEGFA* expression had a significantly higher (p=0.0013) survival rate (more than half of patients are still alive) compared to patients with high *VEGFA* expression (mst 87.5 months) (**Figure 4**).

## 4 Discussion

The results of the presented study showed pRCC having higher HIF-1α protein expression than ccRCC. Previous studies in ccRCC showed that HIF-1α expression is frequently upregulated due to mutations or inactivation of the *VHL* gene, which is a common feature in this subtype (5, 14). Loss of pVHL function results in the stabilization and accumulation of HIF-1α, even under normoxic conditions, leading to increased HIF-1α protein expression and activation of its downstream target genes (15). In contrast to ccRCC, pRCC and chRCC typically have intact *VHL* genes and lower HIF-1α expression compared to ccRCC (16). As shown above, our samples had more tumor necrosis present, which, by itself presents a strong hypoxia inducer, which is a plausible explanation for higher HIF-1α immunohistochemical expression. Furthermore, pRCC may exhibit increased HIF-1α expression due to factors other than *VHL* mutations (17).

There was no significant correlation between HIF-1α protein expression and patients’ age or tumor size. Some studies have also not found a significant correlation between HIF-1α expression and age in ccRCC (18, 19). On the other hand, other studies have reported a correlation between HIF-1α expression and age in ccRCC, suggesting that HIF-1α expression may be higher in older patients with ccRCC compared to younger patients (20). The rationale behind this correlation is that as people age, there may be cumulative cellular damage and alterations in the tumor microenvironment, including hypoxia, which could lead to increased HIF-1α expression. The relationship between HIF-1α and age could be influenced by various factors, including tumor stage, grade, and other molecular features. Overall, while some studies suggest a correlation between HIF-1α expression and age in ccRCC, more research is needed to establish a clear and consistent association. Regarding tumor size, previous studies showed a correlation between HIF-1α expression and renal tumor size, particularly in clear cell renal cell carcinoma (ccRCC) (21). Studies have shown that HIF-1α expression tends to be higher in larger tumors since HIF-1α plays a crucial role in the regulation of cellular responses to hypoxia (low oxygen levels) (22). As the tumor grows, it may outgrow its blood supply, leading to areas of hypoxia within the tumor mass. In response to hypoxia, HIF-1α becomes stabilized and accumulates, leading to increased HIF-1α expression in larger tumors. It’s important to note that the correlation between HIF-1α and tumor size is not uniform across all renal cancer subtypes or other types of solid tumors (14, 23). The relationship may vary depending on the tumor’s specific genetic and molecular characteristics, or, as it is in our case, the presence of tumor necrosis.

Our study revealed no significant difference among tumor types regarding RT-qPCR analysis of *HIF1A* mRNA. This could be attributed to tumor sample number, given that previous works defined higher *HIF1A* mRNA in ccRCC, than in pRCC or chRCC (24–26), however, the differential expression analysis we performed on TCGA studies with a larger sample size than any of the referenced studies has also demonstrated no significant differences in *HIF1A* expression between the analyzed tumor groups. In ccRCC, *HIF1A* is extensively studied and plays a central role in tumor development and progression (27–29). The dysregulation of *HIF1A* in ccRCC contributes to the hypervascular nature of the tumor and its aggressive behavior. In contrast to ccRCC, the role of *HIF1A* in pRCC is less well-defined and may vary among different subtypes. Studies have shown that *HIF1A* expression in pRCC is generally lower than in ccRCC (28, 30). The molecular characteristics of pRCC, including the expression of *HIF1A* and its downstream targets, can differ depending on the specific subtype of pRCC (30, 31). *HIF1A* expression in chRCC is typically lower compared to ccRCC since it is usually associated with intact *VHL* gene function (32, 33).

We used the Hscore method to determine VEGF-A expression in tumor cells. We used the Hscore method for the interpretation of immunohistochemical expression because of its advantage over other methods in data quantification. While many other studies quantify the proportion of positive cells so that a range of proportions is given a specific discrete value, the Hscore method takes the specific proportion of positive cells with a given staining intensity and uses that exact continuous value to calculate the final score (34, 35). In our study, VEGF-A protein expression measured by the Hscore was significantly higher in female patients compared to males, while there were no significant differences regarding nuclear grade or the presence of necrosis. When analyzing histological subtypes, pRCC had the highest VEGF-A Hscore, but the results weren’t significantly different. There was also no significant correlation between VEGF-A protein expression and patients’ age or tumor size. The study by Song et al. showed that pRCC had higher VEGF-A expression compared to ccRCC, which was similar to our results (10). Interestingly, our differential expression analysis revealed significantly higher expression of VEGFA in ccRCC compared to both pRCC and chRCC. Additionally, pRCC had a significantly lower expression than chRCC. The same results were obtained by Situ et al. when analyzing databases that contain the same data as the database we used in our study but with different tools for gene expression analysis (36).

Unlike ccRCC, where *VEGFA* overexpression is more prevalent and consistently associated with poorer prognosis (37), the relationship between VEGF expression and pRCC outcomes appears to be more complex. A study has reported higher VEGF expression in pRCC tumors associated with adverse clinicopathological features, such as larger tumor size, higher stage, and lymph node involvement, suggesting a potential association with aggressive tumor behavior (10). Our study is in accordance with those findings, having shown that in our pRCC population with low *VEGFA* expression, overall survival was significantly prolonged. We found no significant difference in survival regarding *VEGFA* expression in ccRCC and chRCC, unlike other studies (38, 39), however, they analyzed the protein expression, not mRNA expression. While a study by Minardi et al. found that high HIF-1α expression was associated with worse prognosis in ccRCC (19), we have not found any significant difference in RCC patient survival regarding *HIF1A* expression.

While this retrospective study included only samples from a single institution, and even though the sample number was limited, it is important to note that the Pathology Department at the University Hospital in Split is a reference center for the region of Dalmatia (Croatia) and parts of the neighboring Republic of Bosnia and Herzegovina. Also, given the recent update to the renal cell cancer classification, our study is the first, to the best of our knowledge, to implement the novel classification, thus clearly separating ccRCC from other types, specifically eosinophilic-like ccRCC that were known to be misclassified previously as chRCC, while only classifying as chRCC those tumors, that after extensive immunohistochemical analysis fulfilled criteria for chRCC, ensuring exclusion of other oncocyte neoplasms of low malignant potential (3).

## Data availability statement

The datasets presented in this study can be found in online repositories. The names of the repository/repositories and accession number(s) can be found in the article/**Supplementary Material**.

## Ethics statement

The studies involving humans were approved by University Hospital Centre Split, Croatia, Hospital Ethics Committee (class: 500-03/20-01/09, approval number: 2181-147-01/06/M.S.-20-09, approval date: 13 May 2020). The studies were conducted in accordance with the local legislation and institutional requirements. Written informed consent for participation was not required from the participants or the participants’ legal guardians/next of kin in accordance with the national legislation and institutional requirements.

## Author contributions

AS: Conceptualization, Formal Analysis, Investigation, Resources, Writing – original draft, Writing – review & editing. JK: Conceptualization, Methodology, Writing – review & editing. MO: Conceptualization, Formal Analysis, Methodology, Software, Visualization, Writing – review & editing. NK: Methodology, Resources, Software, Writing – review & editing. MV: Investigation, Methodology, Resources, Writing – review & editing. PD: Software, Supervision, Visualization, Writing – review & editing. ST: Conceptualization, Investigation, Methodology, Resources, Software, Writing – original draft, Writing – review & editing.
