# Supplementary material for: Differential expression of HIF1A and its downstream target VEGFA in the main subtypes of renal cell carcinoma and their impact on patient survival
Source: Front Oncol. 2023 Nov 20;13:1287239. doi: 10.3389/fonc.2023.1287239 (PMC10694430; doi:10.3389/fonc.2023.1287239)
Supplement: Supplementary file 1 [file Table_1.docx]

Supplementary Table S1. GAPDH median cycles and interquartile ranges for the clear cell (ccRCC), chromophobe (chRCC), and papillary (pRCC) renal cell carcinoma group. The numbers indicate that the cohorts are comparable.

|  | **ccRCC** | **chRCC** | **pRCC** |
| --- | --- | --- | --- |
| Median | 32.94 | 31.67 | 34.88 |
| 25th percentile | 31.91 | 27.765 | 30.99 |
| 75th percentile | 33.59 | 33.6075 | 35.45 |
